# Strategy to Improve Diagnostic Performance of Hepatic Steatosis Grading Using Ultrasound: Combination of Qualitative and Quantitative Approaches

**DOI:** 10.3390/medicina62081467

**Published:** 2026-07-29

**Authors:** Abdusattorov Shavkat Shokirjon ugli, Sunyoung Lee, Ja Kyung Yoon, Jae Seung Lee, Seung-seob Kim

**Affiliations:** 1 Department of Medical Radiology-2, The Multidisciplinary Children’s Clinic of Tashkent State Medical University, Tashkent State Medical University, Tashkent 100109, Uzbekistan; shavkatmu@gmail.com; 2 Department of Radiology, Research Institute of Radiological Science, Severance Hospital, Yonsei University College of Medicine, Seoul 03722, Republic of Korea; carnival0126@gmail.com (S.L.); jkyoon87@yuhs.ac (J.K.Y.); 3 Department of Internal Medicine, Yonsei University College of Medicine, Seoul 03722, Republic of Korea

**Keywords:** non-alcoholic fatty liver disease, ultrasonography, image interpretation, computer-assisted, sensitivity and specificity

## Abstract

*Background and Objectives*: To propose an integrated qualitative and quantitative US strategy for improving the diagnostic accuracy of hepatic steatosis (HS) grading. *Materials and Methods*: We retrospectively identified patients at a tertiary hospital who underwent B-mode US examination, attenuation imaging (ATI), hepatorenal index (HRI), and liver biopsy on the same day between 2023 and 2024. B-mode liver US images were independently reviewed by three radiologists with varying levels of experience. Each radiologist qualitatively assessed the degree of HS using a three-tier grading system: (1) no HS, (2) mild HS, and (3) moderate/severe HS. Median attenuation coefficient (AC) and HRI values were recorded. Liver biopsy pathology reports were used as the reference standard. We evaluated four strategies integrating qualitative assessment with ATI or HRI and compared their diagnostic performance. *Results*: Based on B-mode US, accuracies for detecting HS by the three radiologists were 87.0%, 87.0%, and 86.0%, respectively; for moderate/severe HS, the values were 79.0%, 76.0%, and 71.0%. ATI and HRI alone yielded diagnostic accuracies of 83.0% and 81.0%, respectively, for any HS and 67.0% and 53.0%, respectively, for moderate/severe HS. The highest accuracy was achieved using a strategy that retained the qualitative grade for one-grade discrepancies and assigning the intermediate grade only for two-grade discrepancies between the AC-based grade and the qualitative grade (93.0%, 87.0%, and 90.0% for any HS; 80.0%, 76.0%, and 74.0% for moderate/severe HS). *Conclusions*: The proposed integration strategy, which retained the qualitative grade for one-grade discrepancies and assigned the intermediate grade only for two-grade discrepancies, improved or maintained diagnostic performance, regardless of the radiologist’s level of experience.

## 1. Introduction

Metabolic dysfunction-associated steatotic liver disease (MASLD), formerly known as non-alcoholic fatty liver disease (NAFLD), has become the leading cause of chronic liver disease worldwide [1]. MASLD represents a spectrum of disease severity, ranging from simple hepatic fat accumulation to progressive inflammatory and fibrotic changes [1]. When hepatic steatosis (HS) is accompanied by hepatocellular inflammation and ballooning, the condition progresses to metabolic dysfunction-associated steatohepatitis (MASH) [1]. Persistent inflammation in MASH drives the development of hepatic fibrosis, which can further progress to cirrhosis and eventually liver failure and/or hepatocellular carcinoma [1].

Historically, lifestyle modification through dietary intervention and increased physical activity has been the mainstay of treatment for MASLD [1]. However, the recent Food and Drug Administration approval of resmetirom, a thyroid hormone receptor beta-selective agonist, marks a significant advancement in pharmacologic therapy [2]. Resmetirom improves all key histopathological features of MASH, including steatosis, hepatocellular ballooning, and lobular inflammation, as well as the fibrosis [2]. More recently, agents with diverse mechanisms—including incretin mimetics such as semaglutide and agonists targeting the fibroblast growth factor 21 receptor or peroxisome proliferator-activated receptors—have continued to receive approval [3]. As therapeutic options continue to diversify and advance, the precise evaluation of treatment response has become increasingly important. Although biopsy remains the gold standard for assessing the histopathologic factors, global interest in non-invasive diagnostic methods continues to grow due to the lifelong management required for patients with MASLD.

Magnetic resonance proton density fat fraction is the most well-established non-invasive technique for quantifying HS and is widely regarded as a surrogate for biopsy in many clinical trials [4]. However, as an MRI-based technology, its high cost and limited availability pose significant challenges—particularly given the high prevalence of MASLD. In this context, ultrasound (US)-based techniques offer a practical and accessible alternative, owing to their lower cost, broad availability, and lack of ionizing radiation.

Traditionally, US-based assessment of HS has relied on the qualitative interpretation of B-mode images. More recently, quantitative techniques—primarily those based on the attenuation coefficient and/or backscatter coefficient—have been introduced, enabling more objective measurements with improved interobserver agreement [5]. In practice; however, these two approaches often produce conflicting findings. Some authors, based on earlier studies that reported the low sensitivity of qualitative methods for diagnosing mild HS [6,7], may argue that only results derived from quantitative analysis should be considered [8]. However, as the quality of B-mode US images has continuously improved with advances in US technology, their diagnostic performance has also progressively increased. One meta-analysis demonstrated that the sensitivity for diagnosing mild HS was higher in studies published after 2010 than in those published before 2010 [8]. Another more recent study reported that radiologists’ overall impression provided more accurate grading of HS than quantitative analysis [9]. Thus, qualitative assessment by experienced radiologists still has the potential to improve the final accuracy of diagnosing and grading HS. However, there is no consensus on how exactly to integrate both qualitative and quantitative results to maximize the diagnostic accuracy.

Importantly, neither qualitative nor quantitative assessment provides a perfectly accurate estimate of the underlying degree of HS. Both approaches are subject to measurement variability and imperfect classification. Accordingly, substantial discordance between qualitative and quantitative assessments may reflect increased uncertainty regarding the underlying disease severity, suggesting that reliance on either extreme classification alone may be suboptimal. The purpose of our study was to propose a simple and practical approach for integrating qualitative and quantitative analyses in grading HS during routine US examinations. We hypothesized that a grade adjustment strategy—reassigning discordant results to an intermediate category—would improve diagnostic performance.

## 2. Materials and Methods

This study was approved by the institutional review board (IRB) of Severance Hospital, and the requirement for written informed consent was waived given the retrospective nature of the study (IRB number: 4-2025-0379, approval date: 23 May 2025). The completed STARD 2015 checklist is provided as Supplementary File S1.

### 2.1. Patients

We retrospectively identified patients at a tertiary care center who underwent both liver US examination using Aplio i800 (Canon Medical System, Canon Medical Systems Corporation, Otawara, Japan) and liver biopsy under US guidance with Arietta 850 (Hitachi, Tokyo, Japan) on the same day between 2023 and 2024. Exclusion criteria were as follows: (1) Lack of quantitative analysis (attenuation imaging [ATI]) for HS during the US examination, (2) Absence of HS grading information in the pathology report. The patients’ age, sex, and body mass index (BMI) were documented.

### 2.2. Qualitative Analysis

Liver US images were independently reviewed by three board-certified radiologists. Their years of experience were as follows: reader 1 (S.S.K., 10 years after board certification; 7 years in gastrointestinal radiology), reader 2 (S.L., 9 years; 9 years), and reader 3 (J.K.Y., 5 years; 5 years). Each reader was instructed to grade HS using a three-tier system: no steatosis, mild steatosis, or moderate/severe steatosis, according to their own judgment and criteria. Only the liver US images were provided; no additional clinical information or data regarding the overall distribution of HS grades in the study population were disclosed to the readers.

### 2.3. Quantitative Analysis

The liver US images and corresponding radiology reports were retrospectively reviewed, and attenuation coefficient (AC, expressed in dB/cm/MHz) values—including the median and the ratio of the interquartile range (IQR) to the median—were documented. At our institution, if the IQR-to-median ratio exceeded 0.3, additional measurements were obtained to replace outlier values. Because various AC cutoff values for HS grading have been proposed in the literature, we adopted the median of those reported values, setting the AC cutoff values at 0.63 for mild HS and 0.69 for moderate/severe HS [10].

The hepatorenal index (HRI) was measured from the US image in which the right lobe of the liver and the right kidney were visualized simultaneously. Circular ROIs of identical size were placed in the liver and kidney at equal depth from the US probe. The HRI was calculated by dividing the mean pixel echogenicity value of the liver by that of the kidney. When the renal cortex and medulla were distinguishable, the ROI was positioned entirely within the cortex. Care was also taken to avoid inclusion of rib shadow and, for the hepatic ROI, large intrahepatic vessels. The HRI was measured up to three times, and the median value was used for statistical analysis. As with AC, various cutoff values for HRI have been proposed. We adopted 1.30 for mild HS [11] and 1.52 for moderate/severe HS [12,13,14,15,16,17,18], calculated as the median of the cutoff values proposed in previous studies (Supplementary Table S1).

### 2.4. Combination of Qualitative and Quantitative Analyses

**Figure 1:**
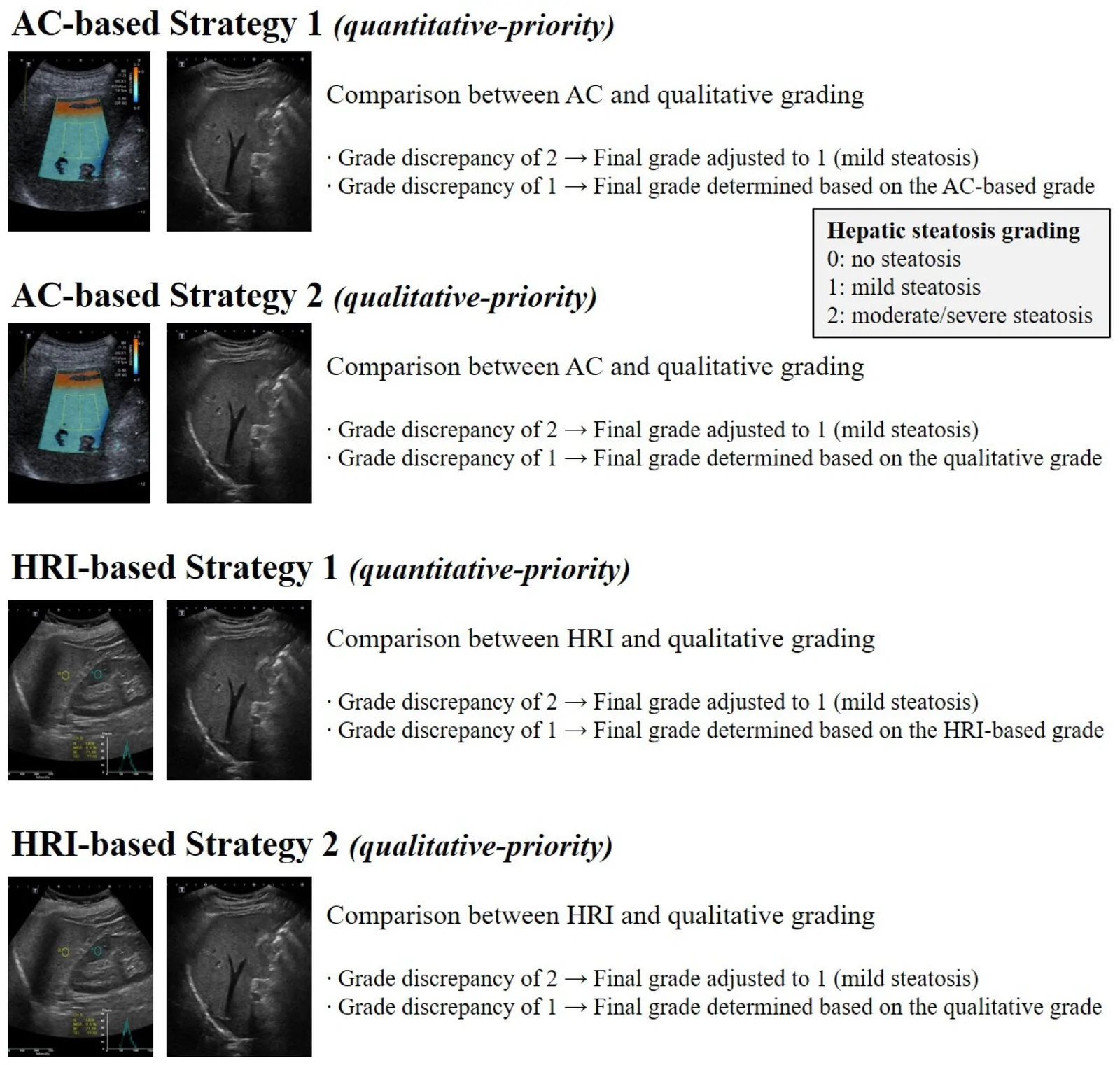
Schematic illustration of the four integration strategies combining qualitative and quantitative analyses. In all four strategies, a two-grade discrepancy between qualitative and quantitative assessments was resolved by assigning the intermediate grade. For a one-grade discrepancy, Strategy 1 (quantitative-priority) adopted the quantitative result, whereas Strategy 2 (qualitative-priority) adopted the qualitative result.

We devised two strategies to integrate qualitative and quantitative analyses and compared their diagnostic performance. In both strategies, discrepancies of two grades (i.e., no HS vs. moderate/severe HS) were resolved by assigning the intermediate grade as the final grade (i.e., mild HS). In the quantitative-priority strategy (Strategy 1), when the two methods differed by one grade, the quantitative result was adopted as the final grade. In the qualitative-priority strategy (Strategy 2), when they differed by one grade, the qualitative result was adopted as the final grade. As quantitative analysis included both ATI and HRI, we ultimately evaluated the diagnostic performance of four integration approaches: AC-based Strategy 1, AC-based Strategy 2, HRI-based Strategy 1, and HRI-based Strategy 2 (Figure 1).

### 2.5. Reference Standard

The HS grade stated in the liver biopsy pathology reports was considered the reference standard. HS grading was defined according to the percentage of steatotic hepatocytes as follows: 0–5%, no HS; 5–33%, mild HS; 33–66%, moderate HS; and >66%, severe HS [19]. For consistency with US-based analyses, moderate and severe HS were merged into a single category, moderate/severe HS. Histopathologic diagnosis and liver fibrosis grade were additionally collected whenever available [20]. Pathology reports that used former terms such as NAFLD were reclassified according to the revised nomenclature of MASLD [21].

### 2.6. Statistical Analysis

Differences in median AC and HRI values among the three HS groups were assessed using the Kruskal–Wallis test, with post hoc pairwise comparisons performed by Dunn’s test with Holm adjustment. The sensitivity, specificity, and accuracy of HS grading were calculated for qualitative analyses (performed independently by three readers), quantitative analyses (ATI and HRI), and combined analyses, respectively. Pairwise comparisons of diagnostic performance between analyses were performed using McNemar’s test for paired binary outcomes. Inter-reader agreement was assessed using Fleiss’ kappa across three readers. In addition, pairwise weighted Cohen’s kappa (quadratic weights) was computed to reflect the ordinal nature of the 3-grade HS scale. The strength of agreement was interpreted according to the guidelines proposed by Landis and Koch: kappa values of <0.00 indicated poor agreement, 0.00–0.20 slight, 0.21–0.40 fair, 0.41–0.60 moderate, 0.61–0.80 substantial, and 0.81–1.00 almost perfect agreement. All statistical analyses were conducted with R software (version 4.5.1).

## 3. Results

### 3.1. Patients

**Figure 2:**
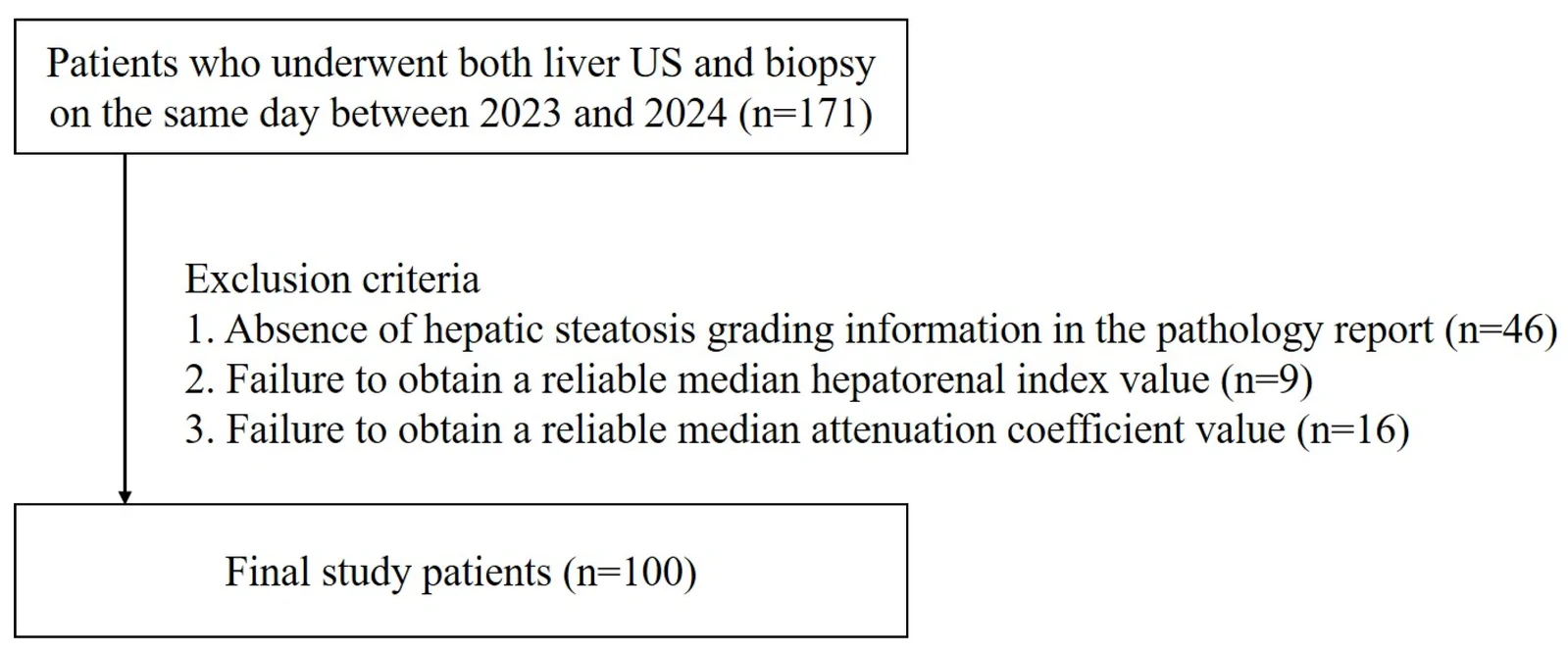
The patient selection flow.

The patient selection flow is summarized in Figure 2. A total of 171 patients who underwent both liver US and biopsy on the same day between 2023 and 2024 were identified. Among them, 46 patients whose pathology reports lacked information on hepatic steatosis grading were excluded. The US images of the remaining 125 patients were then reviewed, and 25 patients were further excluded because reliable HRI or AC measurements could not be obtained. Consequently, 100 patients were finally enrolled in the study.

Their demographic characteristics and liver histopathologic findings are summarized in Table 1. MASH was the most common diagnosis, accounting for 49% of the patients (22 men [45%] and 27 women [55%]). Considering the full steatotic liver disease spectrum, 63% of patients (32 men [51%] and 31 women [49%]) were classified as having a steatotic etiology. Among the remaining patients, 18% (3 men [17%] and 15 women [83%]) showed findings of chronic hepatitis and 8% (4 men [50%] and 4 women [50%]) showed cirrhosis. HS grading revealed no HS in 16% of patients (6 men [38%] and 10 women [62%]), mild HS in 59% (23 men [39%] and 36 women [61%]), and moderate/severe HS in the remaining 25% (15 men [60%] and 10 women [40%]). Liver fibrosis grading demonstrated grade 1 in 44% of patients (23 men [52%] and 21 women [48%]), grade 2 in 19% (7 men [37%] and 12 women [63%]), and grades 3 and 4 in 10% each (grade 3: 1 man [10%] and 9 women [90%]; grade 4: 6 men [60%] and 4 women [40%]).

### 3.2. Inter-Reader Agreement in Qualitative Analysis by Three Readers

Inter-reader agreement among the three radiologists for HS grading was fair (Fleiss’ kappa = 0.265, *p* < 0.001). In pairwise comparisons, readers 1, 2, and 3 demonstrated fair to substantial agreement with one another (weighted Cohen’s kappa = 0.306, 0.312, and 0.675; Table 2).

### 3.3. Diagnostic Performance of Qualitative Analysis

The diagnostic performance of qualitative assessments by each of the three readers for predicting any HS (i.e., mild or moderate/severe HS) and moderate/severe HS is summarized in Table 3. Overall, all three readers showed higher sensitivity than specificity in predicting any HS, whereas specificity exceeded sensitivity in predicting moderate/severe HS. However, the absolute values of sensitivity and specificity varied considerably among the readers. In contrast, accuracy was generally comparable across readers 1, 2, and 3 (87.0%, 87.0%, and 86.0%, respectively). For predicting moderate/severe HS, accuracy ranged from 71.0–79.0% among the three readers.

### 3.4. Distribution of AC and HRI According to HS Grades

**Figure 3:**
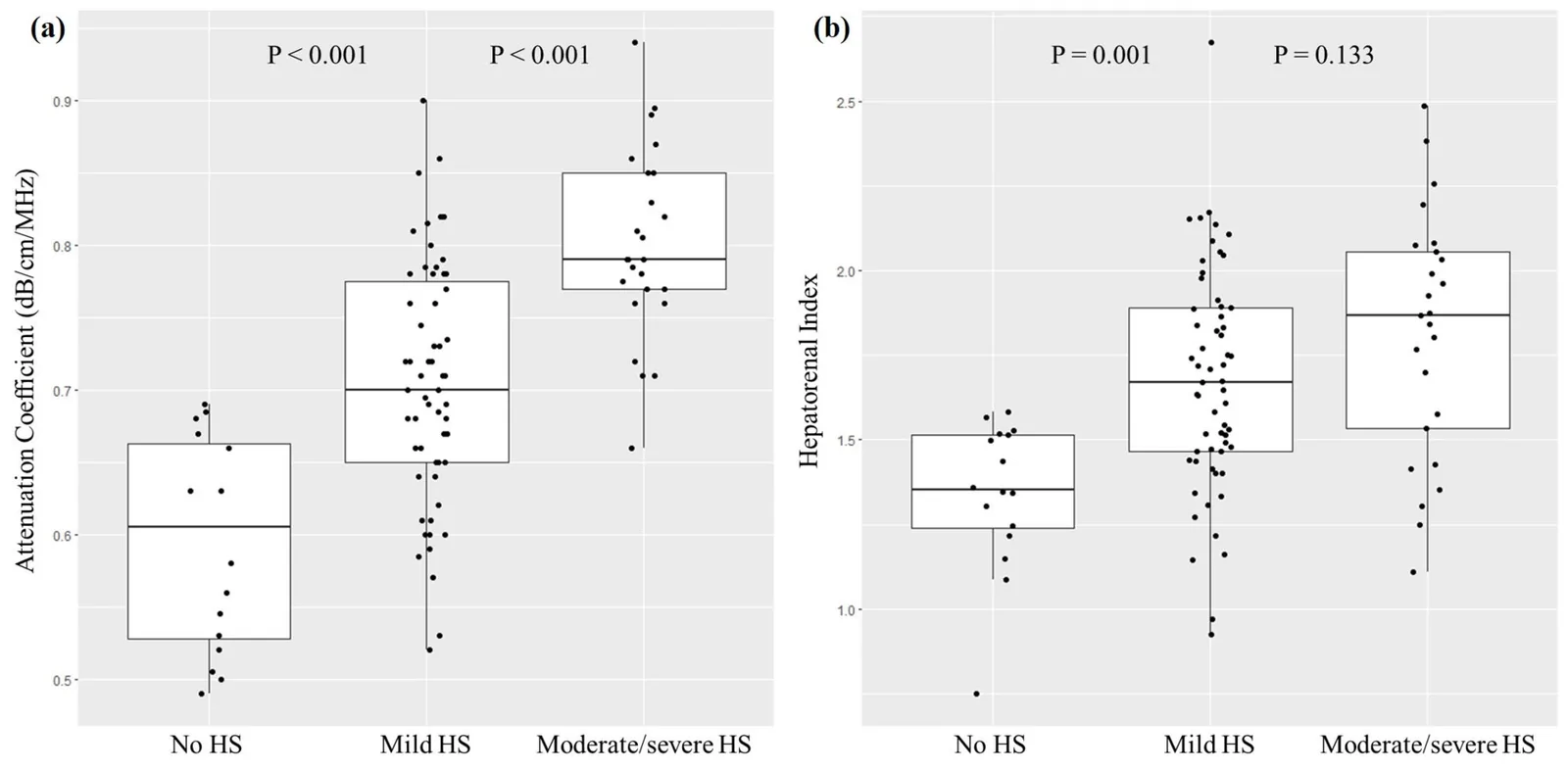
Box-whisker plots showing the distribution of attenuation coefficients (**a**) and hepatorenal indices (**b**) according to hepatic steatosis grading. Dots represent individual observations. Boxes indicate the IQR, with the horizontal line indicating the median; whiskers extend to the most extreme values within 1.5 times the IQR. (**a**) Median attenuation coefficient values were 0.61 (IQR: 0.53–0.66) for no HS, 0.70 (IQR: 0.65–0.78) for mild HS, and 0.79 (IQR: 0.77–0.85) for moderate/severe HS. Post hoc analysis showed significant differences between all adjacent groups (all *p* < 0.001). (**b**) Median hepatorenal index values were 1.35 (IQR: 1.24–1.51) for no HS, 1.67 (IQR: 1.46–1.89) for mild HS, and 1.87 (IQR: 1.54–2.05) for moderate/severe HS. Post hoc analysis showed a significant difference between no and mild HS (*p* = 0.001), but not between mild and moderate HS (*p* = 0.133). HS, hepatic steatosis, IQR, interquartile range.

AC values differed significantly across HS grades (*p* < 0.001; Figure 3a). Median AC values were 0.61 (interquartile range [IQR]: 0.53–0.66) for no HS, 0.70 (IQR: 0.65–0.78) for mild HS, and 0.79 (IQR: 0.77–0.85) for moderate/severe HS. Post hoc analysis showed significant differences between all adjacent groups (all *p* < 0.001).

HRI values showed overall differences among HS grades (*p* = 0.001; Figure 3b). Median HRI values were 1.35 (IQR: 1.24–1.51) for no HS, 1.67 (IQR: 1.46–1.89) for mild HS, and 1.87 (IQR: 1.54–2.05) for moderate/severe HS. Post hoc analysis showed a significant difference between no and mild HS (*p* = 0.001), but not between mild and moderate HS (*p* = 0.133).

### 3.5. Diagnostic Performance of Quantitative Analysis

The diagnostic performance of the two quantitative analyses (ATI and HRI) is summarized in Table 3. Both indices demonstrated generally high sensitivity but relatively low specificity for predicting any HS (ATI: sensitivity, 86.9%; specificity, 62.5%; HRI: sensitivity, 90.5%; specificity, 31.3%). A similar trend was observed when predicting moderate/severe HS (ATI: sensitivity, 96.0%; specificity, 57.3%; HRI: sensitivity, 76.0%; specificity, 45.3%). Accuracy was consistently higher with ATI than with HRI for both any HS (83.0% vs. 81.0%) and moderate/severe HS (67.0% vs. 53.0%).

### 3.6. Diagnostic Performance of Combined Qualitative and Quantitative Analysis

The diagnostic performance of the combined qualitative and quantitative analyses is summarized in Table 3. For all three readers, AC-based Strategy 2 yielded the highest accuracy for predicting any hepatic steatosis (93.0%, 87.0%, and 90.0%, respectively). For the prediction of moderate/severe HS, the highest accuracy was achieved with AC-based Strategy 2 in all three readers (80.0%, 76.0%, and 74.0%, respectively). The numbers of patients reclassified by AC-based Strategy 2 relative to qualitative assessment alone are presented in Supplementary Table S2. Diagnostic performance using cohort-specific Youden-derived cutoff values for AC and HRI is presented in Supplementary Table S3.

The diagnostic performance of AC-based Strategy 2 was statistically compared with its individual components, AC and qualitative grading (Table 4). The statistical significance of the changes in diagnostic performance metrics achieved with AC-based Strategy 2 varied among the readers.

### 3.7. Subgroup Analysis

A subgroup analysis according to BMI (<25 kg/m^2^ vs. ≥25 kg/m^2^) was performed for the prediction of any HS (Supplementary Table S4). Because only three patients with BMI < 25 kg/m^2^ had moderate/severe HS, subgroup analysis for moderate/severe HS was not performed. Overall, diagnostic accuracy tended to be lower in patients with BMI < 25 kg/m^2^ and higher in those with BMI ≥ 25 kg/m^2^ for both qualitative and quantitative analyses. The magnitude of this difference varied across methods, with HRI showing the largest difference between BMI subgroups, followed by qualitative assessment by readers 2 and 3, whereas ATI and reader 1 were less affected. Among the integration strategies, AC-based Strategy 2 remained the best-performing approach in patients with BMI ≥ 25 kg/m^2^. In contrast, in patients with BMI < 25 kg/m^2^, AC-based Strategy 1 achieved higher diagnostic accuracy than AC-based Strategy 2 for readers 2 and 3.

## 4. Discussion

When a significant discrepancy existed between qualitative and quantitative HS grades, adopting an intermediate grading strategy led to improved or maintained diagnostic performance, regardless of the radiologist’s level of experience. Diagnostic accuracy was higher when AC, rather than HRI, was used as the quantitative method. When the discrepancy was minor, relying on the qualitative grade yielded the highest diagnostic accuracy.

Since the introduction of AC-based quantification for HS, a substantial body of research has evaluated its diagnostic performance, with many studies reporting higher accuracy compared with conventional qualitative assessment using B-mode imaging alone [8]. In particular, the limited sensitivity of qualitative B-mode evaluation for detecting mild HS has often been cited as a key limitation [22,23]. However, this comparison should be interpreted with caution, as most studies evaluating qualitative assessment were published before 2010, whereas studies assessing AC-based quantitative methods have been published predominantly since 2019 [8]. Given the continuous advancements in US hardware and image reconstruction algorithms, re-evaluation of this conventional perception appears warranted.

In a recent meta-analysis, the pooled sensitivity for detecting mild HS was 0.75 for AC-based methods and 0.69 for conventional B-mode-based assessment [8]. Notably, when the analysis was restricted to studies published after 2017, the sensitivity of conventional B-mode-based assessment for mild HS diagnosis ranged from 0.73 to 0.83 [8]. In our study, although the overall diagnostic accuracy of qualitative assessment varied according to radiologist experience, the sensitivity for detecting mild HS was higher in three of the three radiologists compared with AC-based quantification alone. Furthermore, integrating qualitative and quantitative assessments improved both overall diagnostic performance for HS and grading accuracy. This improvement was generally observed regardless of radiologist experience. These findings are consistent with a recent study published in 2024, which reported that qualitative imaging features demonstrated superior diagnostic performance compared with ATI alone, and that the radiologist’s overall impression—integrating qualitative and quantitative information—outperformed either approach considered independently [9].

Previous studies have reported that a higher BMI may adversely affect the accuracy and reproducibility of AC-based techniques [4]. Our subgroup analysis likewise suggested that the optimal integration strategy may differ according to BMI. Specifically, in patients with BMI < 25 kg/m^2^, a strategy placing relatively greater weight on quantitative assessment (AC-based Strategy 1) appeared to perform better for some readers, whereas in patients with BMI ≥ 25 kg/m^2^, AC-based Strategy 2 consistently remained the best-performing approach. Although these findings are hypothesis-generating because of the limited sample size, they raise the possibility that a BMI-tailored integration strategy could further improve diagnostic performance and warrants investigation in future studies.

Nevertheless, our findings should be interpreted with caution. Quantitative evaluation must ultimately remain the cornerstone of non-invasive diagnosis and treatment response monitoring for HS. Qualitative assessment, regardless of technological advancements, is inherently prone to inter-reader variability and cannot detect subtle fluctuations in hepatic fat content that do not manifest as a change in HS grade. The clinical significance of our study lies in demonstrating that current ATI technology has not yet reached ideal technical maturity; therefore, in cases of discrepancy between visual grading and AC values, the quantitative measurement should not be assumed to be inherently more accurate.

Our study has several limitations. First, HS grading was performed via retrospective review of stored PACS images by radiologists other than the original operators. This design was necessary to evaluate inter-reader variability within a retrospective framework; however, real-time qualitative grading by original operators, who can utilize dynamic scanning, might yield higher accuracy than the static image review performed in this study. Second, although the integrated strategies (particularly AC-based Strategy 2) demonstrated numerical improvements in diagnostic performance compared to individual methods, these improvements did not reach statistical significance in all instances or for all readers. This may be attributed to our relatively modest sample size, which potentially limited the statistical power to detect significant differences. In addition, the optimal integration strategy was identified and evaluated within the same study cohort, which may have resulted in an optimistic estimate of its diagnostic performance. Independent external validation is therefore required before broader clinical implementation. Another limitation is that serum-based biomarkers reflecting metabolic dysfunction were not incorporated into our analysis [24,25,26]. Future studies integrating qualitative and quantitative ultrasound findings with serum-based biomarkers may further improve diagnostic performance. Future prospective studies with larger sample sizes, in which multiple radiologists independently perform and evaluate US examinations on the same patient, are warranted to address these limitations. Third, the study population was limited to patients with clinically suspected liver disease significant enough to warrant a biopsy. Consequently, our findings may not be directly generalizable to the broader general population.

## 5. Conclusions

In conclusion, our study indicates that integrating qualitative and quantitative US assessments provides a more accurate and reliable method for grading HS than using either approach alone.

## Figures and Tables

**Table 1:** Demographics and liver histopathology of enrolled patients.

	Patients, *n* (% Male)
Age, yrs	49.2 ± 15.8
Male: Female	44:56
BMI, kg/m^2^	28.1 ± 5.4
Histopathologic diagnosis of liver *	
Normal hepatic parenchyma	2 (50)
MASLD without steatohepatitis	9 (56)
MASH	49 (45)
MetALD without steatohepatitis	1 (100)
MetALD with steatohepatitis	3 (100)
Steatohepatitis with unspecified cause	1 (100)
Chronic hepatitis	18 (17)
Cirrhosis	8 (50)
Other pathologies	9 (44)
Hepatic steatosis grade *	
No steatosis (0–5%)	16 (38)
Mild steatosis (5–33%)	59 (39)
Moderate/severe steatosis (>33%)	25 (60)
Liver fibrosis grade *	
Unreported	9 (44)
0	8 (38)
1	44 (52)
2	19 (37)
3	10 (10)
4	10 (60)

* These data were derived from liver pathology reports. BMI, body mass index; MASLD, metabolic dysfunction-associated steatotic liver disease; MASH, metabolic dysfunction-associated steatohepatitis; MetALD, metabolic dysfunction-associated alcohol-related liver disease.

**Table 2:** Pairwise inter-reader agreement in qualitative hepatic steatosis grading.

	Reader 1	Reader 2	Reader 3
Reader 1	–		
Reader 2	0.312	–	
Reader 3	0.675	0.306	–

Values represent weighted Cohen’s kappa (quadratic weights). Only the lower triangle is shown because pairwise kappa values are symmetrical.

**Table 3:** Diagnostic performances of various strategies for predicting hepatic steatosis.

	Predicting Any Hepatic Steatosis			Predicting Moderate/Severe Hepatic Steatosis		
	Sensitivity	Specificity	Accuracy	Sensitivity	Specificity	Accuracy
Qualitative analysis only						
Reader 1	88.1 (79.5–93.4)	81.3 (57.0–93.4)	87.0 (79.0–92.2)	76.0 (56.6–88.5)	80.0 (69.6–87.5)	79.0 (70.0–85.8)
Reader 2	100.0 (95.6–100.0)	18.8 (6.6–43.0)	87.0 (79.0–92.2)	44.0 (26.7–62.9)	86.7 (77.2–92.6)	76.0 (66.8–83.3)
Reader 3	91.7 (83.8–95.9)	56.3 (33.2–76.9)	86.0 (77.9–91.5)	56.0 (37.1–73.3)	76.0 (65.2–84.2)	71.0 (61.5–79.0)
Quantitative analysis only						
AC only	86.9 (78.1–92.5)	62.5 (38.6–81.5)	83.0 (74.5–89.1)	96.0 (80.5–99.3)	57.3 (46.1–67.9)	67.0 (57.3–75.4)
HRI only	90.5 (82.3–95.1)	31.3 (14.2–55.6)	81.0 (72.2–87.5)	76.0 (56.6–88.5)	45.3 (34.6–56.6)	53.0 (43.3–62.5)
Combined qualitative and quantitative analysis						
Reader 1 (AC-based Strategy 1)	88.1 (79.5–93.4)	62.5 (38.6–81.5)	84.0 (75.6–89.9)	96.0 (80.5–99.3)	65.3 (54.1–75.1)	73.0 (63.6–80.7)
Reader 1 (AC-based Strategy 2)	95.2 (88.4–98.1)	81.3 (57.0–93.4)	93.0 (86.3–96.6)	76.0 (56.6–88.5)	81.3 (71.1–88.5)	80.0 (71.1–86.7)
Reader 1 (HRI-based Strategy 1)	91.7 (83.8–95.9)	31.3 (14.2–55.6)	82.0 (73.3–88.3)	76.0 (56.6–88.5)	50.7 (39.6–61.7)	57.0 (47.2–66.3)
Reader 1 (HRI-based Strategy 2)	91.7 (83.8–95.9)	75.0 (50.5–89.8)	89.0 (81.4–93.7)	72.0 (52.4–85.7)	80.0 (69.6–87.5)	78.0 (68.9–85.0)
Reader 2 (AC-based Strategy 1)	86.9 (78.1–92.5)	62.5 (38.6–81.5)	84.0 (74.5–89.1)	96.0 (80.5–99.3)	57.3 (46.1–67.9)	67.0 (57.3–75.4)
Reader 2 (AC-based Strategy 2)	100.0 (95.6–100.0)	18.8 (6.6–43.0)	87.0 (79.0–92.2)	44.0 (26.7–62.9)	86.7 (77.2–92.6)	76.0 (66.8–83.3)
Reader 2 (HRI-based Strategy 1)	91.7 (83.8–95.9)	31.3 (14.2–55.6)	82.0 (73.3–88.3)	76.0 (56.6–88.5)	45.3 (34.6–56.6)	53.0 (43.3–62.5)
Reader 2 (HRI-based Strategy 2)	100.0 (95.6–100.0)	18.8 (6.6–43.0)	87.0 (79.0–92.2)	40.0 (23.4–59.3)	86.7 (77.2–92.6)	75.0 (65.7–82.5)
Reader 3 (AC-based Strategy 1)	90.5 (82.3–95.1)	62.5 (38.6–81.5)	86.0 (77.9–91.5)	96.0 (80.5–99.3)	62.7 (51.4–72.7)	71.0 (61.5–79.0)
Reader 3 (AC-based Strategy 2)	96.4 (90.0–98.8)	56.3 (33.2–76.9)	90.0 (82.6–94.5)	56.0 (37.1–73.3)	80.0 (69.6–87.5)	74.0 (64.6–81.6)
Reader 3 (HRI-based Strategy 1)	90.5 (82.3–95.1)	31.3 (14.2–55.6)	81.0 (72.2–87.5)	76.0 (56.6–88.5)	46.7 (35.8–57.8)	54.0 (44.3–63.4)
Reader 3 (HRI-based Strategy 2)	92.9 (85.3–96.7)	56.3 (33.2–76.9)	87.0 (79.0–92.2)	56.0 (37.1–73.3)	76.0 (65.2–84.2)	71.0 (61.5–79.0)

Data are presented as percentages, with 95% confidence intervals shown in parentheses. Strategy 1 (Quantitative-priority): When qualitative and quantitative analyses differed by two grades, the intermediate grade was assigned. When they differed by one grade, the quantitative result was adopted as the final grade. Strategy 2 (Qualitative-priority): When qualitative and quantitative analyses differed by two grades, the intermediate grade was assigned. When they differed by one grade, the qualitative result was adopted as the final grade. AC, attenuation coefficient; HRI, hepatorenal index.

**Table 4:** Statistical comparison of diagnostic performance among qualitative, quantitative, and combined analyses.

	Predicting Any Hepatic Steatosis			Predicting Moderate/Severe Hepatic Steatosis		
	Sensitivity	Specificity	Accuracy	Sensitivity	Specificity	Accuracy
Reader 1 (AC-based Strategy 2 vs. AC only)	0.02	0.45	0.02	0.07	0.001	0.03
Reader 1 (AC-based Strategy 2 vs. Qualitative only)	0.04	n/a	0.04	n/a	1.00	1.00
Reader 2 (AC-based Strategy 2 vs. AC only)	n/a	0.02	0.48	0.001	<0.001	0.20
Reader 2 (AC-based Strategy 2 vs. Qualitative only)	n/a	n/a	n/a	n/a	n/a	n/a
Reader 3 (AC-based Strategy 2 vs. AC only)	0.01	1.00	0.12	0.004	0.002	0.32
Reader 3 (AC-based Strategy 2 vs. Qualitative only)	0.13	n/a	0.13	n/a	0.25	0.25

Data are *p*-values. Strategy 2 (Qualitative-priority): When qualitative and quantitative analyses differed by two grades, the intermediate grade was assigned. When they differed by one grade, the qualitative result was adopted as the final grade. AC, attenuation coefficient; n/a, not applicable.

## Data Availability

The data presented in this study are available on request from the corresponding author.

## Supplementary Materials

The following supporting information can be downloaded at: https://www.mdpi.com/article/10.3390/medicina62081467/s1, File S1. STARD 2015 Checklist; Table S1. CAP, controlled attenuation parameter [12,13,14,15,16,17,18]; Table S2. Patient reclassification by the proposed AC-based Strategy 2 relative to qualitative assessment alone; Table S3. Diagnostic performances of various strategies for predicting hepatic steatosis using cohort-specific (Youden-derived) cutoffs; Table S4. Diagnostic accuracy for predicting any hepatic steatosis according to BMI subgroup.
