# Supplementary material for: Strategy to Improve Diagnostic Performance of Hepatic Steatosis Grading Using Ultrasound: Combination of Qualitative and Quantitative Approaches
Source: Medicina (Kaunas). 2026 Jul 29;62(8):1467. doi: 10.3390/medicina62081467 (PMC13515070; doi:10.3390/medicina62081467)
Supplement: Supplementary file 1 [file medicina-62-01467-s001.zip › Tables S1-S4.pdf]

Supplementary Table S1. CAP, controlled attenuation parameter.

Reference #	Hepatorenal index cutoff	Definition for moderate/severe hepatic steatosis
[1]	1.25	unspecified
[2]	1.42	Biopsy: >33%
[3]	1.48	Biopsy: >33%
[4]	1.52	Biopsy: >33%
[5]	1.56	CAP: >269dB/m
[6]	1.69	MR spectroscopy: >15%
[7]	1.86	Biopsy: >25%

References

1. Isaksen, V.T.; Larsen, M.A.; Goll, R.; Florholmen, J.R.; Paulssen, E.J. Hepatic steatosis, detected by hepatorenal index in ultrasonography, as a predictor of insulin resistance in obese subjects. *BMC Obes* **2016**, *3*, 39.
2. Moret, A.; Boursier, J.; Houssel Debry, P.; Riou, J.; Crouan, A.; Dubois, M.; Michalak Provost, S.; Aube, C.; Paisant, A. Evaluation of the hepatorenal b-mode ratio and the "controlled attenuation parameter" for the detection and grading of steatosis. *Ultraschall Med* **2022**, *43*, 479–487.
3. Petzold, G.; Lasser, J.; Ruhl, J.; Bremer, S.C.B.; Knoop, R.F.; Ellenrieder, V.; Kunsch, S.; Neesse, A. Diagnostic accuracy of b-mode ultrasound and hepatorenal index for graduation of hepatic steatosis in patients with chronic liver disease. *PLoS One* **2020**, *15*, e0231044.
4. Borges, V.F.; Diniz, A.L.; Cotrim, H.P.; Rocha, H.L.; Andrade, N.B. Sonographic hepatorenal ratio: A noninvasive method to diagnose nonalcoholic steatosis. *J Clin Ultrasound* **2013**, *41*, 18–25.
5. Kozlowska-Petriczko, K.; Wunsch, E.; Petriczko, J.; Syn, W.K.; Milkiewicz, P. Diagnostic accuracy of non-imaging and ultrasound-based assessment of hepatic steatosis using controlled attenuation parameter (cap) as reference. *J Clin Med* **2021**, *10*.
6. Stahlschmidt, F.L.; Tafarel, J.R.; Menini-Stahlschmidt, C.M.; Baena, C.P. Hepatorenal index for grading liver steatosis with concomitant fibrosis. *PLoS One* **2021**, *16*, e0246837.
7. Webb, M.; Yeshua, H.; Zelber-Sagi, S.; Santo, E.; Brazowski, E.; Halpern, Z.; Oren, R. Diagnostic value of a computerized hepatorenal index for sonographic quantification of liver steatosis. *AJR Am J Roentgenol* **2009**, *192*, 909–914.

Supplementary Table S2. Patient reclassification by the proposed AC-based Strategy 2 relative to qualitative assessment alone.

	**Predicting any HS**	**Predicting moderate/severe HS**
**Reader 1**	4 FN -> TP	1 FP -> TN
**Reader 2**	No reclassification	No reclassification
**Reader 3**	4 FN -> TP	3 FP -> TN

Values indicate the number of patients reclassified from an incorrect to a correct classification. HS, he patic steatosis; FN, false negative; TP, true positive; FP, false positive; TN, true negative.

Supplementary Table S3.
Diagnostic performances of various strategies for predicting hepatic steatosis using cohort-specific (Youden-derived) cutoffs.

	**Predicting any HS**			**Predicting moderate/severe HS**		
	Sensitivity	Specificity	Accuracy	Sensitivity	Specificity	Accuracy
Qualitative analysis only						
Reader 1	88.1	81.3	87.0	76.0	80.0	79.0
Reader 2	100.0	18.8	87.0	44.0	86.7	76.0
Reader 3	91.7	56.3	86.0	56.0	76.0	71.0
Quantitative analysis only						
AC only (cutoffs: 0.70 for any HS and 0.76 for moderate/severe HS)	66.7	100.0	72.0	84.0	76.0	78.0
HRI only (cutoffs: 1.61 for any HS and 1.77 for moderate/severe HS)	60.7	100.0	67.0	64.0	70.7	69.0
Combined qualitative and quantitative analysis						
Reader 1 (AC-based Strategy 1)	72.6	100.0	77.0	84.0	80.0	81.0
Reader 1 (AC-based Strategy 2)	91.7	81.2	90.0	76.0	86.7	84.0
Reader 1 (HRI-based Strategy 1)	69.0	100.0	74.0	64.0	70.7	69.0
Reader 1 (HRI-based Strategy 2)	88.1	81.2	87.0	48.0	81.3	73.0
Reader 2 (AC-based Strategy 1)	69.0	100.0	74.0	84.0	76.0	78.0
Reader 2 (AC-based Strategy 2)	100.0	18.8	87.0	44.0	89.3	78.0
Reader 2 (HRI-based Strategy 1)	65.5	100.0	71.0	64.0	70.7	69.0
Reader 2 (HRI-based Strategy 2)	100.0	18.8	87.0	28.0	88.0	73.0
Reader 3 (AC-based Strategy 1)	76.2	100.0	80.0	84.0	77.3	79.0
Reader 3 (AC-based Strategy 2)	92.9	56.2	87.0	56.0	86.7	79.0
Reader 3 (HRI-based Strategy 1)	70.2	100.0	75.0	64.0	70.7	69.0
Reader 3 (HRI-based Strategy 2)	91.7	56.2	86.0	40.0	82.7	72.0

Strategy 1 (Quantitative-priority): When qualitative and quantitative analyses differed by two grades, the intermediate grade was assigned. When they differed by one grade, the quantitative result was adopted as the final grade. Strategy 2 (Qualitative-priority): When qualitative and quantitative analyses differed by two grades, the intermediate grade was assigned. When they differed by one grade, the qualitative result was adopted as the final grade. AC, attenuation coefficient; HRI, hepatorenal index, HS, hepatic steatosis.

Supplementary Table S4.
Diagnostic accuracy for predicting any hepatic steatosis according to BMI subgroup.

	**All patients (n=100)**	**BMI < 25 (n=26)**	**BMI ≥ 25 (n=74)**
Qualitative analysis only			
Reader 1	87.0	84.6	87.8
Reader 2	87.0	61.5	95.9
Reader 3	86.0	65.4	93.2
Quantitative analysis only*			
AC only	83.0	73.1	86.5
HRI only	81.0	53.8	90.5
Combined qualitative and quantitative analysis			
Reader 1 (AC-based Strategy 1)	84.0	73.1	87.8
Reader 1 (AC-based Strategy 2)	93.0	88.5	94.6
Reader 1 (HRI-based Strategy 1)	82.0	53.8	91.9
Reader 1 (HRI-based Strategy 2)	89.0	80.8	91.9
Reader 2 (AC-based Strategy 1)	84.0	73.1	86.5
Reader 2 (AC-based Strategy 2)	87.0	61.5	95.9
Reader 2 (HRI-based Strategy 1)	82.0	53.8	91.9
Reader 2 (HRI-based Strategy 2)	87.0	61.5	95.9
Reader 3 (AC-based Strategy 1)	86.0	76.9	89.2
Reader 3 (AC-based Strategy 2)	90.0	69.2	97.3
Reader 3 (HRI-based Strategy 1)	81.0	53.8	90.5
Reader 3 (HRI-based Strategy 2)	87.0	65.4	94.6

AC, attenuation coefficient; BMI, body mass index; HRI, hepatorenal index.
